# Association of D-dimer, fibrin degradation products, and coagulation parameters with progressive hemorrhagic injury following traumatic brain injury: A retrospective cohort study

**DOI:** 10.1097/MD.0000000000051041

**Published:** 2026-10-09

**Authors:** Nan-Qiong Wu, Jing-Jing Hu, Jie Zhou, Huo-Quan Tang, Bo Li, Xing-Guang Ren, Huang-Suo Wang, Jian Lu

**Affiliations:** a Academy of Medical Sciences, School of Public Health, Shanxi Medical University, Taiyuan, Shanxi, China; b Endoscopy Room, Shanxi Medical University Sixth School of Clinical Medicine (Tai Steel General Hospital), Taiyuan, Shanxi, China; c Department of Neurosurgery, Shanxi Medical University Sixth School of Clinical Medicine (Tai Steel General Hospital), Taiyuan, Shanxi, China.

**Keywords:** coagulopathy, D-dimer, FDP, progressive hemorrhagic injury, traumatic brain injury

## Abstract

This study aimed to evaluate the predictive value of coagulation biomarkers, particularly D-dimer and fibrin degradation products (FDP), in the development of secondary progressive hemorrhagic injury (PHI) following traumatic brain injury (TBI). We conducted a retrospective cohort study based on hospital medical record data. All enrolled TBI patients were divided into a PHI group (n = 51) and a non-PHI group (n = 151) for comparison based on whether PHI occurred during hospitalization. Comprehensive coagulation profiles, including D-dimer, FDP, Prothrombin time, thrombin time, fibrinogen (FIB), and activated partial thromboplastin time (APTT), were analyzed. Multivariate logistic regression was performed to identify independent risk factors. The incidence of PHI was 25.25% (51/202). PHI patients demonstrated significantly higher systolic blood pressure (154.00 mm Hg vs 139.00 mm Hg, *P* = .002) and longer hospital stays (20.00 days vs 13.00 days, *P* = .007). Coagulation analysis revealed lower FIB (2.75 g/L vs 3.04 g/L, *P* = .021) and APTT (28.90 seconds vs 29.90 seconds, *P* = .048), but markedly elevated D-dimer (9162.00 ng/mL vs 3191.00 ng/mL, *P* = .001) and FDP levels (73.77 μg/mL vs 22.19 μg/mL, *P* = .003) in the PHI group. Logistic regression identified Glasgow Coma Scale score as an independent risk factor [adjusted odds ratio = 1.558, 95% confidence interval = 1.056–2.298, *P* = .025] and APTT as a protective factor (odds ratio = 0.937, 95% confidence interval = 0.915–0.959, *P* < .001) of secondary PHI in patients with TBI. Coagulation dysfunction, characterized by elevated D-dimer and FDP and decreased FIB, was significantly associated with PHI development in TBI patients. Multivariable logistic regression revealed that only Glasgow Coma Scale score and APTT remained significant and were independent predictors of secondary PHI.

## 1. Introduction

Traumatic brain injury (TBI) is still a primary cause of death in trauma populations, with studies indicating a 20% mortality rate within 24 hours of injury.^[1]^ The pathophysiology of TBI typically includes substantial bleeding, which alters hemostatic equilibrium and causes fast coagulopathy.^[2]^ According to epidemiological statistics, 40 to 80% of patients with cranial trauma have coagulation dysfunction, which is closely associated with poor outcomes such as cerebral infarction, intracranial hemorrhage, and an increase in surgical intervention rates.^[3–6]^ Progressive hemorrhagic injury (PHI), defined as persistent cerebral bleeding, is a serious consequence of TBI-related coagulopathy.^[7]^ PHI occurs in 30 to 60% of TBI cases and exacerbates neurological degeneration, enhancing the likelihood of permanent impairment (by up to 80%) and death.^[2,8–11]^ These complications highlight the need for early identification of risk factors for PHI, as timely intervention can substantially improve patient survival and functional recovery. Although prior investigations have explored D-dimer and fibrin degradation products (FDP) in TBI-related PHI, few studies focused on the combined predictive performance of coagulation markers alongside Glasgow Coma Scale (GCS) and activated partial thromboplastin time (APTT). In the present study, we assessed these coagulation indices to provide robust evidence for risk stratification of secondary PHI in patients with TBI

## 2. Research objective and methods

### 2.1. Study population and grouping

#### 2.1.1. Study population

A retrospective cohort study of 503 patients with confirmed TBI was enrolled from the Department of Neurosurgery, Shanxi Medical University Sixth School of Clinical Medicine (Tai Steel General Hospital), between January 1, 2020, and December 31, 2023. Patients with spontaneous or nontraumatic intracerebral hemorrhage (n = 154), those taking long-term anticoagulants (e.g., aspirin) or with a history of coagulation disorders (n = 56), 43 patients whose time from injury to hospital presentation exceeded 24 hours, 28 in-hospital deaths, and 20 patients with incomplete medical records containing missing data were excluded. Consequently, 202 patients were included in the final analysis.The sample size for this study was determined based on all accessible cases that fulfilled the inclusion and exclusion criteria within the study period, thereby representing a convenience sample. Missing data were scarce and presumed completely random in this retrospective cohort. Multiple imputation may generate artificial simulated values for core laboratory variables. Cases with missing values for any covariate were excluded from the analytic dataset. Therefore, no missing data were present in the final analysis. The study was approved by the Ethics Committee of Shanxi Medical University Sixth School of Clinical Medicine (Tai Steel General Hospital) [(2021) Ethical Document (K-PJ006)], and all patients were exempted from the right to informed consent.

#### 2.1.2. Inclusion criteria

1. Documented history of traumatic injury;
2. Injuries met the diagnostic criteria outlined in the Fourth Edition of the Guidelines for the Treatment of Severe Craniocerebral Injury^[12]^;
3. Time from injury to hospital admission ≤ 24 hours;
4. Complete clinical records are available for analysis.

#### 2.1.3. Exclusion criteria

1. Spontaneous or nontraumatic intracranial hemorrhage (e.g., cerebral tumors, arteriovenous malformations, ruptured aneurysms);
2. Chronic use of anticoagulants (e.g., aspirin);
3. Preexisting coagulation disorders;
4. Severe polytrauma involving multiple organ systems;
5. Patients whose first computed tomography (CT) examination was not conducted within 2 hours of admission;
6. Patients who died or were transferred to other hospitals during the observation period.

#### 2.1.4. Study groups

Patients were stratified into 2 groups based on the occurrence of progressive intracranial hemorrhage (PHI) during hospitalization. In PHI group (n = 51), there were 42 males (82.4%) and 9 females (17.6%), motor vehicle accidents 18 cases (35.3%), falls 23 cases (45.1%), falls from heights 8 cases (15.7%), and blunt trauma 2 cases (3.9%). In the non-PHI group (n = 151), there were 106 males (70.2%)and 43 females (29.8%), motor vehicle accidents 61 cases (40.4%), falls 56 cases (37.1%), falls from heights 21 cases (13.9%), assaults 7 cases (4.6%), blunt trauma 6 cases (3.9%).

### 2.2. Research methods

#### 2.2.1. Data collection

##### 2.2.1.1. Demographic and clinical parameters

Baseline data were collected at admission, including: demographics (age, gender, systolic blood pressure [SBP]), injury characteristics (mechanism of injury, time from injury to admission), clinical variables (GCS score, use of hemostatic agents, presence of hemorrhage on initial head CT, Surgical condition), hospitalization metrics (duration of hospital stay).

##### 2.2.1.2. Biochemical markers

The following laboratory parameters were recorded at admission: hematological indices (platelet [PLT] count, white blood cell count, absolute neutrophil count), liver function (alanine aminotransferase), coagulation profiles (prothrombin time [PT], APTT, thrombin time (TT), fibrinogen [FIB]), and fibrinolysis markers (FDP, D-dimer).

#### 2.2.2. Diagnostic criteria^[13,14]^

Patients were stratified into 3 severity groups based on GCS scores obtained within 2 hours of admission: mild TBI (GCS 13–14, 134 cases), moderate TBI (GCS 9–12, 17 cases), and severe TBI (GCS ≤ 8, 51 cases).

#### 2.2.3. PHI definition^[15,16]^

PHI was diagnosed if post-injury imaging or surgical findings met one of the following criteria:

1. Imaging progression: second cranial CT revealed ≥ 25% hematoma expansion or new intracranial bleeding compared to baseline CT. The 2 CT examinations were obtained within 24 hours, and both images were independently reviewed by 2 blinded neuroradiologists. Any disagreements were resolved through consensus discussion.
2. Surgical confirmation: intraoperative identification of active intracranial bleeding.

### 2.3. Laboratory detection protocol

Coagulation parameters (PT, FIB, APTT, TT, FDP, D-dimer) were quantified using a BE (BE Automate GmbH, Germany) Series Automated Coagulation Analyzer. Standardized operating procedures were strictly followed:

1. Sample collection: 3.6 mL of elbow venous blood was drawn immediately upon admission and dispensed into 2 anticoagulant vacuum tubes.
2. Centrifugation: samples were mixed gently and centrifuged at 3000 rpm for 10 minutes (4°C) to obtain PLT-poor plasma.
3. Assay execution: plasma samples were analyzed within 2 hours using the coagulometer according to manufacturer protocols.

### 2.4. Statistical analysis

Data analysis was performed using Statistical Package for the Social Sciences 26.0 and parametric and nonparametric tests were applied based on data distribution. Normal distribution was described as Mean ± standard deviation and group comparison using an independent *t*-test. Skewed distribution was described as Median (interquartile range) and group comparison using Mann–Whitney *U* test (2 groups) or Kruskal-Wallis *H* test (≥ 3 groups). Categorical variables were described as frequencies (percentages) (n [%]) and compared using the *χ*^2^ test or Fisher exact test (for small samples),whereas the Benferroni method was utilized for comparisons among subgroups. The difference was statistically significant when *P* < .05. Receiver operating characteristic (ROC) curve analysis was used to verify the predictive value of influencing factors. Multivariate logistic regression was performed to identify independent risk factors, with odds ratio (OR) and 95% confidence interval (CI) reported. Multivariate logistic regression was performed to test the primary hypothesis: whether APTT remained independently associated with PHI after adjustment for GCS grade. This analysis was not designed to build a comprehensive prediction model. Duration of hospital stay was excluded because it is a post-admission outcome rather than a baseline risk factor. When attempting to include all remaining baseline variables with *P* < .05 from univariate analysis (including D‑dimer, FDP, SBP and surgical status), the model failed to converge, likely due to limited PHI events (n = 51). Therefore, a parsimonious model containing only GCS grade and APTT was used.

## 3. Results

### 3.1. Univariate analysis of admission data

Univariate analysis revealed statistically significant differences inSBP, hospitalization duration, surgical condition, and GCS scores between the PHI and non-PHI groups (*P* < .05). Patients in the PHI group exhibited higher SBP (154.00 mm Hg vs139.00 mm Hg) and longer hospital stays (20.00 days vs13.00 days) compared to the non-PHI group, while no significant intergroup differences were observed for other parameters (*P* > .05). Detailed data are presented in Table 1.

**Table 1:** Single-factor analysis of admission data of patients with TBI in the non-PHI group and the PHI group.

Group	Non-PHI group (151 cases)	PHI group (51 cases)		
Mean ± SD			*t* value	*P* value
Age/year	55.60 ± 15.58	58.53 ± 14.92	−1.175	.242
Median (IQR)			*Z* value	*P* value
Systolic blood pressure*/*mm Hg	139.00 (36.00)	154.00 (29.00)	−3.133	.002
Duration of hospital stay*/*d	13.00 (13.00)	20.00 (34.00)	−2.719	.007
Time from injury to admission/h	2.00 (2.00)	2.00 (1.00)	−0.205	.837
n (%)			*χ*^2^ value	*P* value
Gender			2.875	.090
Male	106.00 (70.20)	42.00 (82.40)		
Female	43.00 (29.80)	9.00 (17.60)		
Mechanism of injury			3.103	.554
Motor vehicle accidents	61.00 (40.40)	18.00 (35.30)		
Falls	56.00 (37.10)	23.00 (45.10)		
Falls from heights	21.00 (13.90)	8.00 (15.70)		
Assaults	7.00 (4.60)	0.00 (0.00)		
Blunt trauma	6.00 (4.00)	2.00 (3.90)		
Presence of hemorrhage on initial head CT			0.031	.859
No	12.00 (7.90)	3.00 (5.90)		
Yes	139.00 (92.10)	48.00 (94.10)		
Use of hemostatic agents			2.006	.157
No	30.00 (19.90)	15.00 (29.40)		
Yes	121.00 (80.10)	36.00 (70.60)		
Surgical condition			12.540	< .001
No	121.00 (80.10)	28.00 (54.90)		
Yes	30.00 (19.90)	23.00 (45.10)		
GCS grade			11.147	.030
Light	109.00 (72.20)	25.00 (49.00)		
Medium	8.00 (5.30)	9.00 (17.60)		
Weight	34.00 (22.50)	17.00 (33.30)		

CT = computed tomography, GCS = Glasgow Coma Scale, IQR = interquartile range, n = number of patients, PHI = progressive hemorrhagic injury, SD = standard deviation, TBI = traumatic brain injury.

### 3.2. Coagulation parameter comparison

Significant disparities in FIB, APTT, D-dimer, and FDP levels were identified between the PHI and non-PHI groups (*P* < .05). Specifically, the PHI group demonstrated lower FIB (2.75 g/L vs 3.04 g/L) and APTT (28.90 seconds vs 29.90 seconds) but elevated D-dimer (9162.00 ng/mL vs 3190.00 ng/mL) and FDP (73.77 μg/mL vs 22.19 μg/mL). No significant differences in PLT count, leukocytes, neutrophils, PT, or TT were detected (*P* > .05). See Table 2 for complete results.

**Table 2:** Single-factor analysis of D-dimer, FDP, and coagulation in the non-PHI group and the PHI group.

Group	Non-PHI group (151 cases)	PHI group (51 cases)		
Mean ± SD			*T* value	*P* value
Platelets (10^9^/L)	219.67 ± 59.21	205.43 ± 58.77	1.493	.139
Median (IQR)			*Z* value	*P* value
White blood cells (10^9^/L)	13.12 (6.55)	13.86 (6.26)	−1.248	.212
Neutrophils (10^9^/L)	11.42 (5.92)	11.72 (5.88)	−1.236	.217
ALT (U/L)	17.80 (13.20)	20.40 (21.50)	−0.684	.494
PT*/*s	13.20 (1.80)	13.40 (2.10)	−0.040	.968
FIB (g/L)	3.04 (1.14)	2.75 (1.62)	−2.308	.021
APTT*/*s	29.90 (4.50)	28.90 (4.10)	−1.980	.048
TT/s	14.90 (2.10)	14.70 (2.30)	−0.323	.747
D-D (ng/mL)	3191.00 (9185.00)	9162.00 (26303.00)	−3.322	.001
FDP (μɡ/mL)	22.19 (81.52)	73.77 (139.00)	−3.005	.003

ALT = alanine aminotransferase, APTT = activated partial thromboplastin time, D-D = D-dimer, FDP = fibrinogen degradation products, FIB = fibrinogen, IQR = interquartile range, PHI = progressive hemorrhagic injury, PT = prothrombin time, SD = standard deviation, TT = thrombin time.

### 3.3. Coagulation function across GCS severity groups

Patients were stratified into 3 groups based on GCS scores: mild (134 cases), moderate (17 cases), and severe (51 cases). Kruskal–Wallis analysis revealed statistically significant differences in FIB, TT, D-dimer, and FDP levels among the 3 groups (*P* < .05). Post hoc comparisons showed marked discrepancies between mild and severe TBI subgroups: severe TBI patients had significantly lower FIB (2.67 g/L vs 3.06 g/L) but markedly elevated TT (15.30 seconds vs 14.80 seconds), D-dimer (13684.00 ng/mL vs 2661.50 ng/mL), and FDP (124.60 μg/mL vs 15.86 μg/mL). PT and APTT levels showed no intergroup variation (*P* > .05). Refer to Table 3 for subgroup data.

**Table 3:** Comparison of coagulation function indexes in patients with different degrees of TBI.

GCS grade	PT*/*s	FIB (g/L)	APTT*/*s	TT*/*s	D-D (ng/mL)	FDP (μɡ/mL)
Light	13.15 (1.80)	3.06 (1.22)*	29.80 (4.50)	14.80 (1.80)*	2661.50 (5725.00)*	15.86 (53.65)*
Medium	13.20 (1.50)	2.90 (1.69)	28.90 (2.90)	14.70 (1.80)	12588.00 (37309.00)	66.06 (224.06)
Severe	13.70 (2.30)	2.67 (1.13)*	30.10 (5.00)	15.30 (2.40)*	13684.00 (40687.00)*	124.60 (241.22)*
*H* value	3.346	8.689	4.176	6.154	33.875	37.171
*P*	.188	.013	.124	.046	< .001	< .001

APTT = activated partial thromboplastin time, D-D = D-dimer, FDP = fibrinogen degradation products, FIB = fibrinogen, GCS = Glasgow Coma Scale, PT = prothrombin time, TBI = traumatic brain injury, TT = thrombin time.

* indicates a statistically significant difference between the 2 subgroups (*P* < .05).

### 3.4. ROC analysis of APTT, D-dimer and FIB degradation products for predicting PHI in patients with TBI

As presented in Figure 1. ROC curve analysis was performed to evaluate the predictive performance of APTT, D-dimer, and FDP for PHI among patients with TBI. The corresponding predictive parameters are summarized in Table 4. For D-dimer, the area under the curve (AUC) was 0.656 (95% CI: 0.571–0.740, *P* = .001), with an optimal cutoff value of 6271.00 ng/mL, a sensitivity of 62.70%, and a specificity of 69.50%. For FDP, the AUC was 0.641 (95% CI: 0.558–0.724, *P* = .003), with an optimal cutoff value of 44.85 μg/mL, a sensitivity of 62.70%, and a specificity of 65.60%. Both D-dimer and FDP exhibited moderate predictive performance, supporting their application as auxiliary predictive indicators for PHI in TBI patients. In contrast, APTT yielded an AUC of 0.407 (95% CI: 0.318–0.497, *P* = .048), with an optimal cutoff value of 46.55 seconds, indicating poor predictive ability. Accordingly, APTT is not suitable as an independent predictive marker for PHI in this population.

**Table 4:** ROC analysis of APTT, D-dimer and FDP for predicting PHI in patients with TBI.

Factors	Cutoff value	AUC	*P*	95% CI for AUC	Sensitivity (%)	Specificity (%)
APTT/s	46.55	0.407	.048	0.318–0.497	39.00	99.30
D-D ng/mL	6271.00	0.656	.001	0.571–0.740	62.70	69.50
FDP μɡ/mL	44.85	0.641	.003	0.558–0.724	62.70	65.60

APTT = activated partial thromboplastin time, AUC = area under the curve, CI = confidence interval, D-D = D-dimer, FDP = fibrin and fibrinogen degradation products, PHI = progressive hemorrhagic injury, ROC = receiver operating characteristic, TBI = traumatic brain injury.

**Figure 1.**
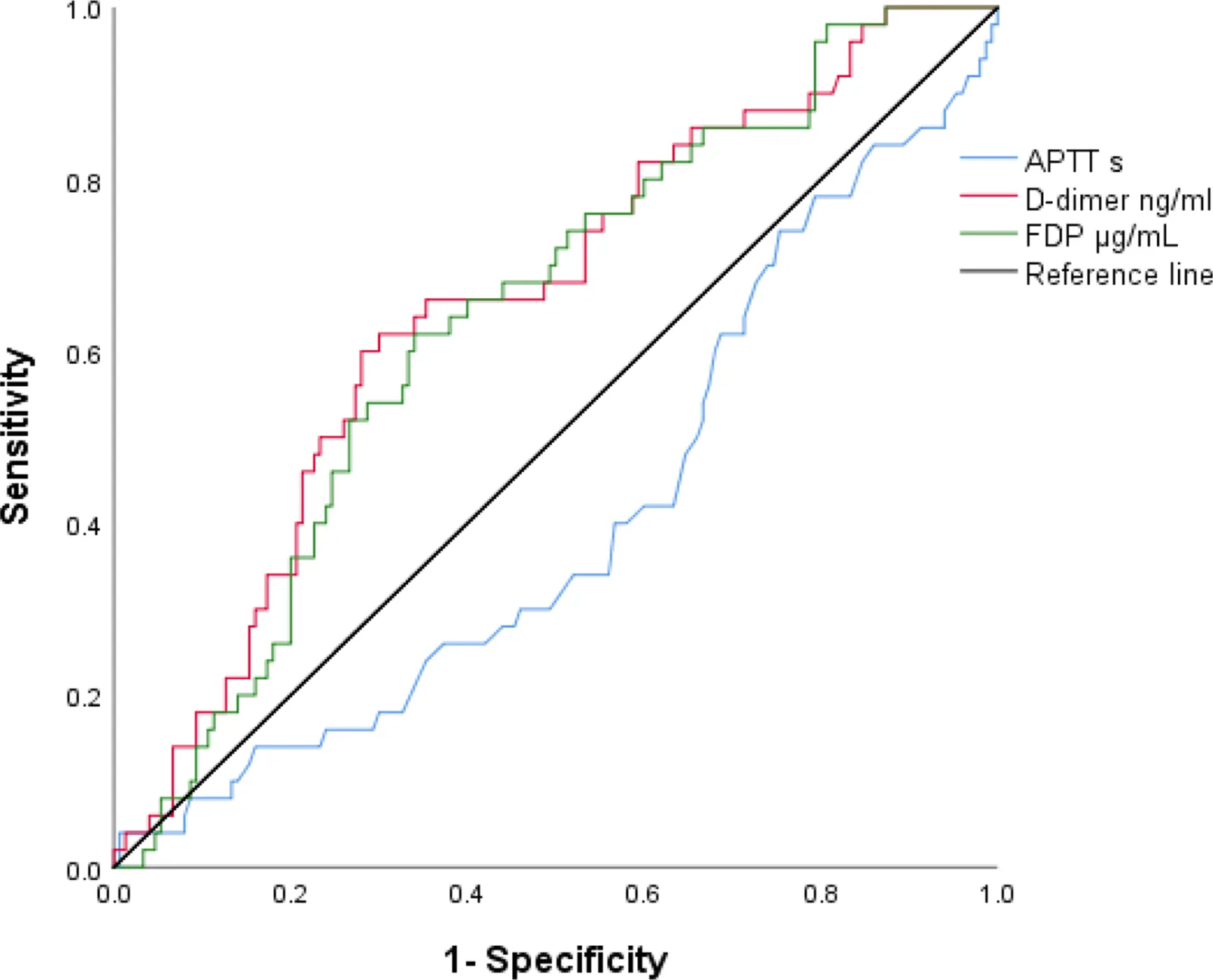
ROC analysis of APTT, D-dimer and FDP for predicting PHI in patients with TBI. APTT = activated partial thromboplastin time, FDP = fibrinogen degradation products, PHI = progressive hemorrhagic injury, ROC = receiver operating characteristic, TBI = traumatic brain injury.

### 3.5. Multivariate logistic regression analysis

Based on the findings from the univariate analysis, the GCS score and APTT value were incorporated into the logistic regression model for further analysis. The GCS score was found to be an independent risk factor for PHI in TBI patients (OR = 1.558, 95% CI = 1.056–2.298, *P* = .025), with each 1-level rise increasing the risk by 1.558 times. In contrast, APTT appeared as a protective factor (OR = 0.937, 95% CI = 0.915–0.959, *P* < .001), with each 1-second increase in APTT reducing PHI risk by 0.937 times. The results are summarized in Table 5.

**Table 5:** Logistic regression analysis of risk factors for secondary PHI in patients with TBI.

Factors	*β*	SE	Walds	*P*	Adjusted OR (95% CI)
GCS	0.443	0.198	4.999	.025	1.558 (1.056–2.298)
APTT	−0.065	0.012	29.325	< .001	0.937 (0.915–0.959)

APTT = activated partial thromboplastin time, CI = confidence interval, GCS = Glasgow Coma Scale, OR = odds ratio, PHI = progressive hemorrhagic injury, SE = standard error, TBI = traumatic brain injury.

## 4. Discussion

Coagulopathy is not only a prevalent consequence of TBI, but it is also closely related to PHI.^[17]^ The regulating mechanisms of the coagulation system are quite complex.^[18]^ After TBI, injured cerebral cortices release tissue factors, which encourage the formation of intracranial thrombosis by depleting clotting components and activating coagulation pathways (such as the protein C system) and inhibitors.^[19,20]^ Excessive tissue factor release overwhelms the brain’s innate thromboplastin activity, causing hypercoagulability and consequent fibrinolytic failure, exacerbating progressive intracranial bleeding.^[21]^

Analysis of 202 TBI patients with secondary PHI in this study showed that PHI was linked to greater SBP and lengthier hospital stays. Clinicians must therefore closely monitor blood pressure fluctuations in the management of TBI. Coagulation parameters (PT, APTT, FIB, and TT) are commonly employed to determine hemostatic state. Clinically, anticoagulant dosages are changed based on PT variations, whereas APTT reflects treatment efficacy. FIB, a crucial thrombin substrate, TT, and a fibrin polymerization indicator all play important roles.^[22,23]^ Our findings revealed substantial differences in APTT, FIB, D-dimer, and FDP levels between the PHI and non-PHI groups.Notably, D-dimer and FDP levels increased with disease severity and may serve as auxiliary markers but are not independent predictors after multivariable adjustment, indicating an association between these coagulation markers and secondary PHI. Targeted monitoring based on coagulation profiles may help optimize clinical management and improve outcomes in patients with TBI. ROC curve analysis revealed that the AUC, 95% CI, *P* value, optimal cutoff value, sensitivity, and specificity of D-dimer and FDP were 0.656 and 0.641, 0.571 to 0.740 and 0.558 to 0.724, 0.001 and 0.003, 6271.00 ng/mL and 44.85 μg/mL, 62.70% and 62.70%, 69.50% and 65.60%, respectively. These findings suggest that abnormal changes in D-dimer and FDP can serve as auxiliary reference indicators for the occurrence of PHI in patients with TBI. The AUC of APTT was 0.407 (95% CI = 0.318–0.497, *P* = .048), with an optimal cutoff value of 46.55 seconds. APTT exhibits poor predictive performance and is not recommended as an independent predictive marker for PHI in patients with TBI. Clinicians should closely monitor the dynamics of coagulation indicators and provide timely, appropriate, and effective treatment to improve patient prognosis and quality of life.

Previous research has shown that coagulopathy in TBI patients increases mortality from 35% (TBI alone) to 85% when paired with coagulation dysfunction, with variable coagulation marker patterns between groups.^[24,25]^ Patients with severe TBI (GCS ≤ 8) showed reduced FIB but significantly higher D-dimer and FDP levels than those with mild-moderate cases (GCS 9–14). This discrepancy is most likely due to significant brain tissue injury that causes thrombin overactivation and consequent coagulopathy. Multivariable logistic regression identified the GCS score as an independent risk factor for PHI (OR = 1.558, 95% CI = 1.056–2.298), whereas APTT was an independent protective factor (OR = 0.937, 95% CI = 0.915–0.959). Notably, this inverse association should be interpreted with extreme caution. Given the observational nature of this study, residual confounding and unmeasured clinical factors may partly account for this protective correlation, and a definite causal relationship cannot be concluded.

The strengths of this study included strict and predefined inclusion and exclusion criteria. Patients receiving anticoagulant medications and those with combined severe extracranial trauma were excluded to minimize selection bias and baseline confounding. All laboratory indicators were detected following unified standardized protocols, which reduced measurement bias. In addition, all CT images and PHI diagnoses were evaluated by independent neuroradiologists who were blinded to patients’ coagulation results, ensuring objective and reliable outcome assessment and lowering diagnostic bias.

Several limitations of this study should also be acknowledged. First, although we adjusted for major known confounding factors, potential unmeasured confounders, including exact injury anatomical location, prehospital emergency management, and critical illness status on admission, may still influence the final results. Second, the relatively small sample size and single-center design may limit the generalizability of our findings. Third, the short follow-up duration may have overlooked delayed hemorrhagic events. Finally, because our dataset only contains ordinal categorical GCS grades (mild/moderate/severe), without original continuous 3 to 15‑point GCS scores. Three ordinal categories cannot generate sufficient cutoff points to construct a reliable ROC curve. ROC analysis for GCS was not performed. Therefore, further large-sample, multicenter, and prospective studies with longer observation periods are required to verify and update our conclusions.

In conclusion, TBI-related coagulopathy is closely associated with secondary PHI. Elevated D-dimer and FDP levels are correlated with disease severity and PHI occurrence, but they do not serve as independent predictive factors after adjusting for confounding variables. In contrast, the GCS score and APTT were identified as independent predictors of secondary PHI in this TBI cohort. Higher GCS scores indicated an increased PHI risk, whereas prolonged APTT was associated with a lower likelihood of PHI. Clinically, close coagulation monitoring and individualized therapeutic strategies are recommended for high-risk TBI patients to facilitate early risk stratification and prevent PHI progression.

## Acknowledgements

We thank the patients whose anonymised data were used for this research. We sincerely thank the Shanxi Provincial Health Commission Plan Project for providing financial support.

## Author contributions

**Conceptualization:** Jie Zhou.

**Data curation:** Nan-Qiong Wu, Jing-Jing Hu.

**Formal analysis:** Nan-Qiong Wu, Jian Lu.

**Funding acquisition:** Huang-Suo Wang.

**Investigation:** Nan-Qiong Wu, Jing-Jing Hu, Huo-Quan Tang.

**Methodology:** Jie Zhou, Xing-Guang Ren.

**Project administration:** Bo Li.

**Resources:** Huang-Suo Wang, Jian Lu.

**Software:** Bo Li.

**Supervision:** Jing-Jing Hu.

**Validation:** Nan-Qiong Wu, Huo-Quan Tang.

**Visualization:** Xing-Guang Ren.

**Writing – original draft:** Nan-Qiong Wu.

**Writing – review & editing:** Jian Lu.
